# Health-related quality of life after surviving intensive care for COVID-19: a prospective multicenter cohort study

**DOI:** 10.1038/s41598-023-45346-2

**Published:** 2023-10-21

**Authors:** Peter Halvorsen, Michael Hultström, Johanna Hästbacka, Ing-Marie Larsson, Rakel Eklund, Filip K. Arnberg, Laura Hokkanen, Robert Frithiof, Ewa Wallin, Lotti Orwelius, Miklós Lipcsey

**Affiliations:** 1 Anesthesiology and Intensive Care Medicine, Department of Surgical Sciences, Uppsala University, Uppsala, Sweden; 2 Integrative Physiology, Department of Medical Cell Biology, Uppsala University, Uppsala, Sweden; 3 Department of Perioperative and Intensive Care Medicine, Helsinki University Hospital, and Helsinki University, Helsinki, Finland; 4 Department of Anesthesiology and Intensive Care, Tampere University Hospital and Tampere University, Tampere, Finland; 5 Department of Medical Sciences, National Centre for Disaster Psychiatry, Uppsala University, Uppsala, Sweden; 6 Department of Psychology and Logopedics, Faculty of Medicine, University of Helsinki, Helsinki, Finland; 7 Departments of Intensive Care, Linköping University Hospital, Linköping, Sweden; 8 Biomedical and Clinical Sciences, Linköping University, Linköping, Sweden; 9 Hedenstierna Laboratory, Department of Surgical Sciences, Uppsala University, Uppsala, Sweden; 10 Department of Anesthesia and Intensive Care, Uppsala University, Akademiska sjukhuset, Ing 70, 751 85 Uppsala, Sweden

**Keywords:** Infectious diseases, Quality of life, Outcomes research

## Abstract

In survivors of severe coronavirus disease 2019 (COVID-19) incomplete mental and physical recovery may considerably impact daily activities and health-related quality of life (HRQoL). HRQoL can be evaluated with the RAND-36 questionnaire, a multidimensional instrument that assesses physical and mental aspects of health in eight dimensions. The objective was to investigate HRQoL in intensive care patients previously treated for COVID-19 at three Nordic university hospitals, in a prospective multi-center cohort study. HRQoL was measured using RAND-36, 3–9 months after discharge from intensive care units (ICU). One hospital performed a second follow-up 12 months after discharge. A score under the lower limit of the 95% confidence interval in the reference cohorts was considered as significantly reduced HRQoL. We screened 542 and included 252 patients. There was more than twice as many male (174) as female (78) patients and the median age was 61 (interquartile range, IQR 52–69) years. Hypertension was the most common comorbidity observed in 132 (52%) patients and 121 (48%) patients were mechanically ventilated for a median of 8 (IQR 4–14) days. In RAND-36 physical functioning, physical role functioning, general health (*p* < 0.001 for all) and social functioning (*p* < 0.05) were below reference, whereas bodily pain, emotional role functioning and mental health were not. In a time-to-event analysis female sex was associated with a decreased chance of reaching the reference HRQoL in the physical function, bodily pain and mental health dimensions. Higher body mass index was found in the physical functioning dimension and hypertension in the physical functioning, vitality and social functioning dimensions. Similar results were seen for diabetes mellitus in general health, vitality and mental health dimensions, as well as pulmonary illness in the physical role functioning dimension and psychiatric diagnosis in the social functioning dimension. Mechanical ventilation was associated with a decreased likelihood of achieving reference HRQoL in the bodily pain and physical functioning dimensions. Patients treated in an ICU because of COVID-19 had lower HRQoL 3–9 months after ICU discharge than 95% of the general population. Physical dimensions were more severely affected than mental dimensions. Female sex and several comorbidities were associated with a slower rate of recovery.

*Study registration*: clinicaltrials.gov: NCT04316884 registered on the 13th of March 2020, NCT04474249 registered on the 29th of June 2020 and NCT04864938 registered on the 4th of April 2021.

## Introduction

During the coronavirus disease 2019 (COVID-19) pandemic, many patients presented with severe illness requiring hospitalization and intensive care. Over six million people died worldwide, in Finland there were close to nine thousand deaths and in Sweden almost twenty-four thousand in COVID-19^1^. In those surviving severe COVID-19 mental and physical recovery has been reported to occur at varying degrees and following different trajectories. Incomplete recovery, e.g. persistent dyspnea, fatigue and cognitive symptoms, may have considerable impact on daily activities and health-related quality of life (HRQoL)^2–10^ even affecting the families of intensive care unit (ICU) survivors^11^.

Studies have shown that COVID-19 and intensive care negatively impact short- and long-term HRQoL^12–15^. The mechanisms of incomplete recovery in patients with mild to severe COVID-19 and post-intensive care are multifaceted^7,16–19^. One study^20^ suggested that invasive ventilation affects HRQoL at a 1-year follow-up compared to a mixed hospitalized cohort, including other patients treated at an ICU with diagnoses other than COVID-19. Moreover, in Sweden, the RAND-36 tool is widely used to assess HRQoL after intensive care as a standard in The Swedish Intensive Care Registry. Yet, large reports on HRQoL assessed with RAND-36, have been scarce in patients treated in an ICU with COVID-19.

Our main hypothesis is that COVID-19 patients treated in ICU have decreased HRQoL compared to the normal range of the general population as a consequence of severe illness and the impact of intensive care. Thus, this study aimed to measure HRQoL in a large group of COVID-19 patients who received intensive care.

In this study we investigated patients treated in intensive care at three Nordic university hospitals and registered their HRQoL 3 to 6 months after discharge from ICU using the RAND-36^21^. We chose to measure the patients HRQoL with the generic instrument RAND-36 since it together with 36-Item Short form Survey^22^ (SF-36) a similar instrument, is the most widely used instruments used to measure HRQoL^23^. The availability of reference populations in both Sweden^23^ and Finland^24^ as well as the fact that it measures and separates different aspect of mental and physical health. As a secondary endpoint, we investigated how demographic factors, previous comorbidity and invasive ventilation, a proxy for more demanding intensive care, affected HRQoL in a time-to-event analysis.

## Materials and methods

We conducted a prospective multi-center cohort study on HRQoL in patients surviving intensive care for COVID-19 infection. Patients were included at tertiary care hospitals in Uppsala and Linköping in Sweden, and in Helsinki, Finland. Included patients were admitted to ICU 3rd of April 2020 to the 11th of August 2021 and followed up between 9th of July 2020 until December 8th, 2022.

Patients ≥ 18 years of age treated at an ICU due to COVID-19 in any of the hospitals described above were eligible for inclusion with informed consent. Patients unable to complete the follow-up protocol or give informed consent were not included. See supplement for details concerning inclusion and exclusion of the different sites. The follow-up was planned for 3 to 6 months after ICU discharge. The Uppsala site performed a second follow-up 12 months after discharge.

The follow-up was conducted by sending the RAND-36 questionnaires to the participants in advance. The questionnaires were collected when the participants visited an investigator or they were submitted by post after the visit. Some patients received and returned the questionnaires only through the post.

We collected data on sex, age, body mass index (BMI) and clinical parameters (e.g., ICU length of stay, mechanical ventilation and ventilator time) at intensive care admission. The medical records were screened for prior comorbidity for the following diagnosis groups: hypertension, other cardiovascular diagnoses, diabetes mellitus, pulmonary diagnoses, malignancy and psychiatric diagnoses. We did correlation analysis on preliminary data showing strong correlation between the factors concerning severity of disease. The correlations between the collected comorbidities, age and sex were weaker. Therefore, we only used presence of mechanical ventilation as the disease severity parameter, and included comorbidities, age and sex as covariates that are commonly used in intensive care studies.

### Scoring instrument

RAND-36 was used to assess physical, mental and social aspects of health. The instrument contains 36 items divided into eight subscales to measure health status: physical function, physical role function, bodily pain, general health, vitality, social function, mental health and emotional role function. The first four dimensions measure physical health and the last four mental and social health. The scores on all sub-scales are transformed to a scale from 0 (the worst score) to 100 (the best score). The RAND-36 was developed to reflect the part of the World Health Organization’s definition of health that pertains to self-reports of physical, mental and social well-being and functional ability^21,25^. It has been translated and validated for the Swedish^26^ and Finnish populations^24^. A score in a dimension below the lower limit of the 95% confidence interval (CI) of the respective reference population was considered significantly below normal HRQoL.

### Statistics and calculations

Cohort size was not pre-determined, but the different sites aimed to include all patients receiving care at an ICU for COVID-19. Histogram inspection revealed that data were not normally distributed. Consequently, we used non-parametric methods to analyze the data. In the primary outcomes missing data was 2.4% at maximum. Missing data in the RAND-36 dimensions were imputed with the median of that dimension in the calculation of IQR. For other variables, missing values were omitted.

Reference level of quality of life (QoL) was defined as having a RAND-36 score within the 95% confidence interval (CI) of a Swedish reference cohort^23^ for the Swedish observations, respective a Finnish reference cohort^24^ for the Finnish observations, in the specified dimension. The reference cohorts above were not exposed to COVID-19 since data were collected prior to the COVID-19 pandemic. We used a two-sided binomial test to determine whether HRQoL in our cohort was below the lower limit of the 95% CI of the relevant reference cohort.

We assumed HRQoL to be decreased during intensive care and defined recovery as a RAND-36 score above the lower limit of the 95% CI of the reference material at follow-up. Patients were called to follow-up at specific times but actual time to follow-up showed a substantial variation in time. This variation in follow-up times allows for a continuous analysis of recovery using time-to-event analysis assuming the variation in time to follow-up is not determined by the severity of symptoms. Thus, we analyzed the outcome predictors in a time-to-event analysis using a Cox proportional hazard model. Because there was multicollinearity between the length of stay in the ICU, mechanical ventilation and days mechanically ventilated, we chose to use treatment with mechanical ventilation as our variable for the severity of COVID-19 in this model. We also performed both the binominal test and the time to event analysis as a sensitivity analysis against the Swedish reference cohort (see Supplement).

R version 4.1.2 (The R Foundation for Statistical Computing, Vienna, Austria; https://www.r-project.org) was used for calculations with package ggplot2 3.3.5, survival 3.3–1, survminer 0.4.8 and survivalAnalysis 0.3.0. The level of statistical significance was set at *p* < 0.05.

### Ethical approval

The National Ethical Review Agency approved the study in Uppsala (2020-02697 with amendments 2020-03629, 2020-05758, 2021-02205, 2022-01115-02), Linköping (2021-01283 and 2022-07064-02) and a local ethics board of the Helsinki University hospital district in Helsinki (HUS-1949–2020). The Declaration of Helsinki and its subsequent revisions were followed.

### Consent to participate

Written informed consent was obtained from the patients.

## Results

**Figure 1:**
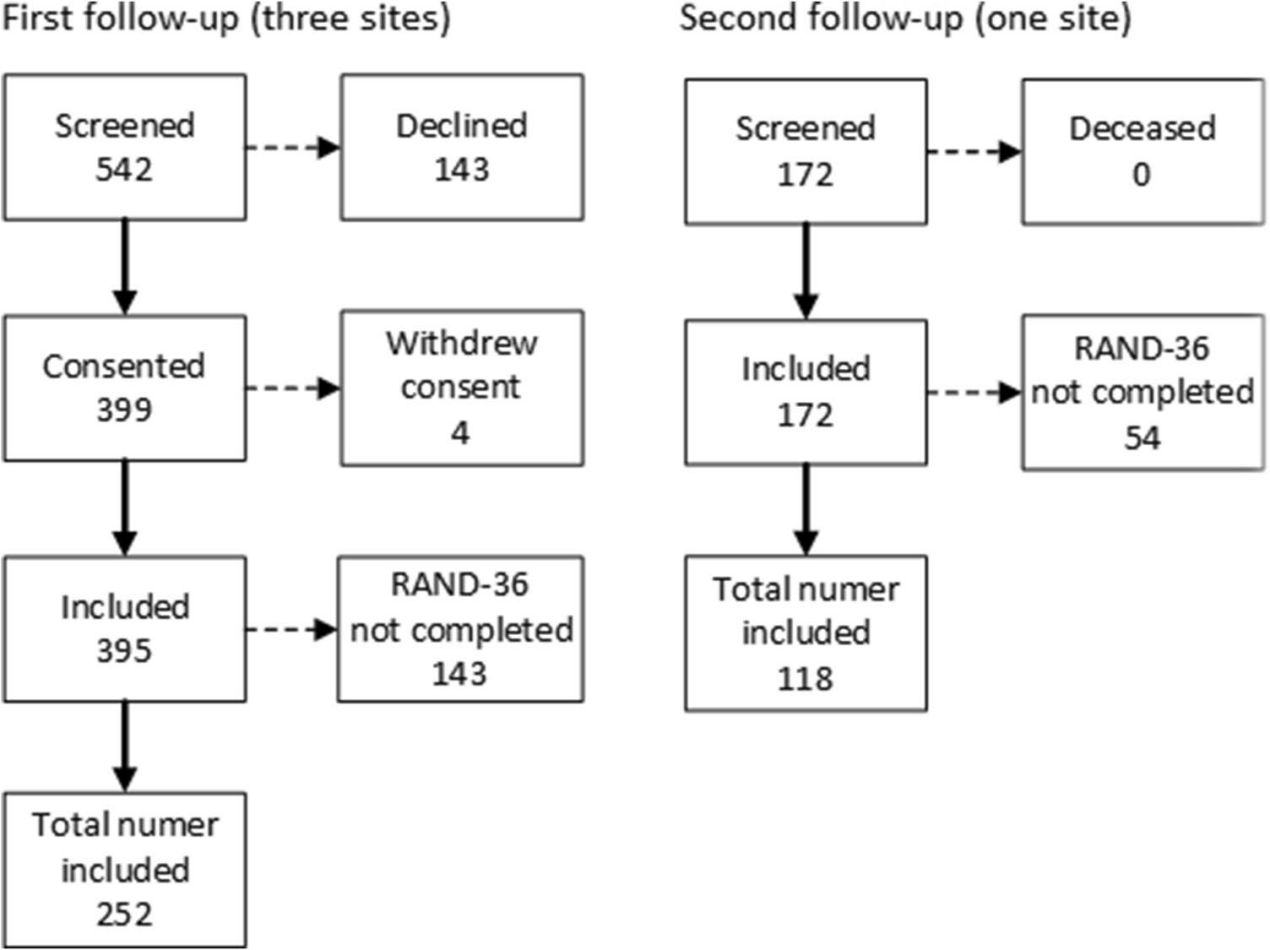
Flowchart of study patients for the first and second follow-up.

We screened 542 patients for participation. Of these, 143 declined, 4 withdrew consent and 143 did not answer, leaving a final sample of 252 (46%) participants in the first follow-up. One site conducted a second follow-up inviting 172 participants, of whom 118 (69%) returned the RAND-36 questionnaires (see Fig. 1). The first follow-up was performed at a median of 157 (IQR 116–180) days and the second at a median of 428 (IQR 369–501) days.

Comorbidity and clinical characteristics of patients are presented in Table 1. There was more than twice as many male (174) as female (78) patients, and the median age was 61 (IQR 52–69) years. Most patients had some comorbidity, with hypertension as the most common. Almost 50% of the patients were mechanically ventilated when treated for COVID-19. The median BMI at ICU admission was 29.7 (IQR 26.7–33.7) kg/m^2^.

**Table 1:** Characteristics of patients who completed the RAND-36 questionnaire at the first follow-up.

Characteristic n = 252	Median	n% or IQR
Sex (female)	78	31%
Married*	176	78%
Age (years)	61	52–69
BMI (at admission to ICU)	30	27–34
Hypertension	132	52%
Other cardiovascular diagnoses	46	18%
Diabetes	60	24%
Pulmonary diagnosis	52	21%
Psychiatric diagnosis	17	7%
Malignancy	18	7%
Days in ICU	9	6–16 (range 1–52)
Days with mechanical ventilation (all)	0	0–9
Mechanically ventilated	121	48%
Days with mechanical ventilation (ventilated)	8	4–14 (range 1–41)

Data are presented as n (%) or median and IQR unless otherwise stated.

*IQR* interquartile range, *ICU* intensive care unit, *BMI* body mass index.

*Married or living with a partner.

**Figure 2:**
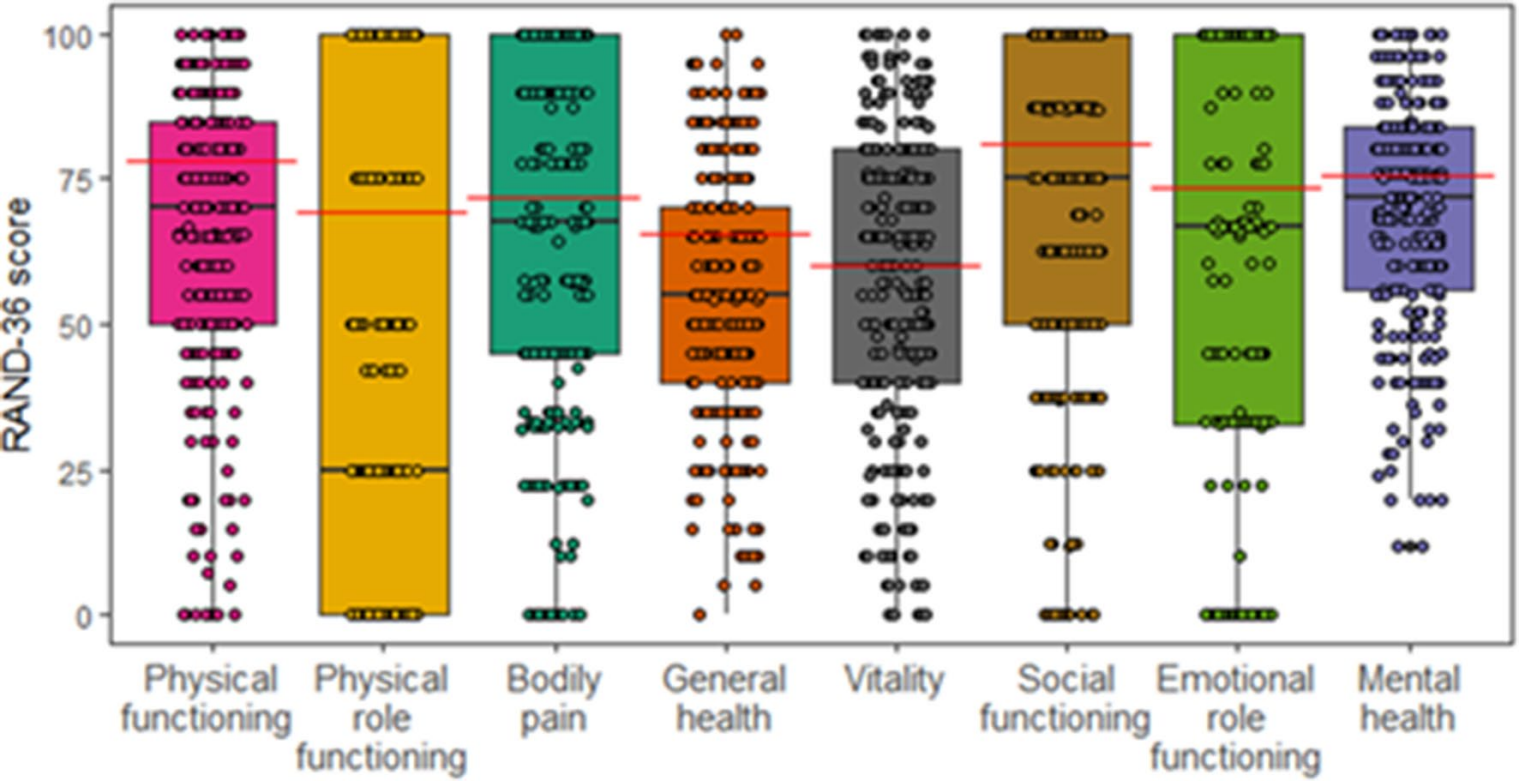
Dimension and RAND-36 scores at the first follow-up with the lower limit of the Swedish reference data 95% CI^23^ in red for respective dimensions. The black lines show median and the boxes shown IQR.

The RAND-36 scores at the first follow-up are presented in Fig. 2. At the first follow-up, 3 of 4 physical dimensions were significantly lower than the lower limit of the 95% CI compared to the respective countries reference cohort (Table 2)^23,24^. For mental dimensions, 1 of 4 was lower compared to the respective reference cohort (Table 2). We also performed the analysis with the Swedish reference cohort’s lower 95% CI limit as a sensitivity analysis, returning similar results (se Supplement).

**Table 2:** Our RAND-36 data at first follow-up vs the lower limit of the 95% CI in a Swedish reference material^23^ for the Swedish observations and a Finnish reference material^24^ for the Finnish observations.

Dimension	95% CI for 0.5	*P*-value	n below ref	N total
Physical functioning	0.290–0.409	< 0.001	157	246
Physical role functioning	0.298–0.421	< 0.001	158	246
Bodily pain	0.380–0.506	0.077	140	251
General health	0.269–0.389	< 0.001	167	248
Vitality	0.418–0.546	0.611	128	247
Social functioning	0.365–0.492	< 0.05	142	248
Emotional role functioning	0.407–0.534	0.375	132	249
Mental health	0.394–0.522	0.203	134	247

Two-sided binomial test was used, CI below 0.5 (represents the binominal cut-of point) indicates decreased health compared to references.

*CI* confidence interval.

**Figure 3:**
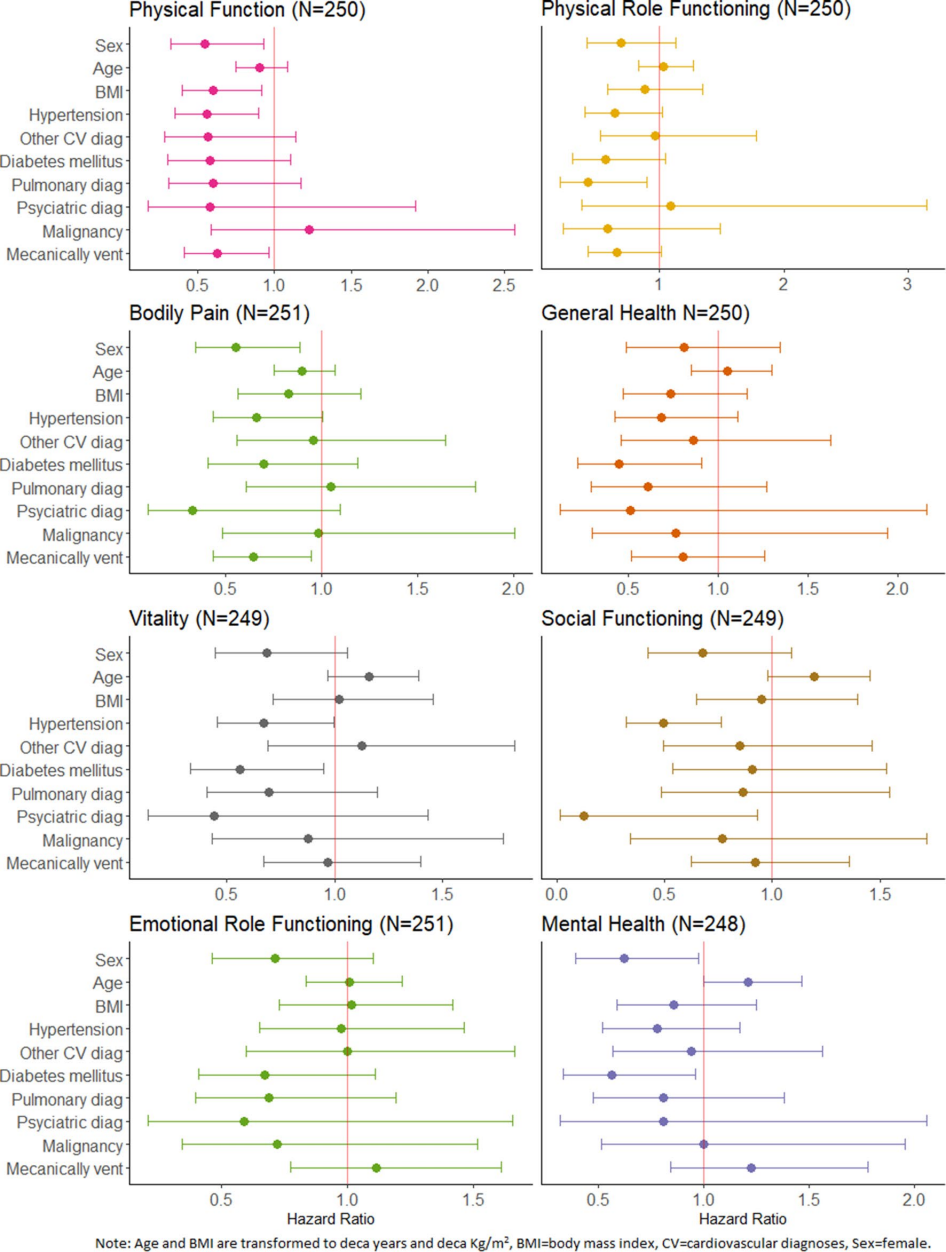
Hazard ratios for the RAND-36 dimensions, including all observations in reference to the respective countries reference cohort’s lower 95% CI limit^23,24^ as endpoint.

In the time-to-event analysis, performed on the complete cohort (first and second follow-up), we assessed predictors of regaining HRQoL (Fig. 3) in reference to respective national reference cohort’s lower 95% CI limit. Female sex was associated with a decreased chance of regaining HRQoL in the physical functioning, bodily pain and mental health dimensions and increasing BMI in the physical functioning dimension. Concerning comorbidity before ICU admission, hypertension was associated with a decreased chance of regaining HRQoL in the physical functioning, vitality and social functioning dimensions, diabetes mellitus in the general health, vitality and mental health dimensions, pulmonary illness in the physical role functioning dimension and psychiatric diagnosis in the social functioning dimension. Mechanical ventilation during the ICU stay was associated with a decreased chance of ameliorating HRQoL in the physical functioning and bodily pain dimensions.

## Discussion

The main finding of this study is that patients who received intensive care for COVID-19 perceived significantly lower levels of HRQoL in the physical dimensions of the RAND-36 (3 of 4 dimensions) compared to the previously collected reference populations^23,24^, suggesting reduced physical capacity. One mental dimension was significantly reduced, implying that social and mental health are affected, but potentially less so than the physical dimensions. Other studies on HRQoL in the general population during the pandemic are scarce and we found none utilizing RAND-36^25^ or SF-36^22^ in the studied countries. However, one Swedish study with the less detailed instruments, Visual Analog Scale (VAS) and EQ-5D-5L^27^ reported also a reduction of HRQoL in the general Swedish population during the COVID-19 pandemic.We found that females had a numerically lower recovery rate in all dimensions, significant for physical functioning, bodily pain and mental health. Similar data have been reported in a previous study^28^ comparing sexes in a post-COVID-19 cohort and an ECMO survivor cohort^29^ where women perceived lower levels of HRQoL. However, women also perceived generally lower RAND-36 scores in all dimensions in the Swedish reference material^23^, suggesting that female sex affects HRQoL. In a previous study in post-COVID-19 patients^30^ an increased risk of onset of new pain after severe COVID-19 and decreased HRQoL were reported, although there were no sex differences. The skewed proportions between the sexes with double the number of males are in line with other studies describing patients admitted to the ICU for COVID-19^31^as well as proportions diagnosed with COVID-19^20^. It has been reported that males are struck harder by the disease ^32,33^ however not fully understood.

Hypertension has been reported to be a risk factor for ICU admission for COVID-19^31^, and its negative impact on HRQoL has been shown in a population-based study in Sweden^34^. In the current study hypertension negatively affected the regaining of health in both the physical dimension; physical functioning as well as the mental dimensions; vitality and social functioning, which is consistent with the finding that hypertension is associated with more severe illness^31^. However, hypertension is closely related to age, which may bias this interpretation. Thus, more severe COVID-19 in hypertensive patients could reduce HRQoL and slow recovery.

In this study we report that mechanical ventilation was a negative factor in restoring HRQoL for both physical function and bodily pain in an ICU cohort. This finding suggests that the severity of illness affects the recovery in HRQoL even in critically ill patients, which may be mediated partly through mechanical ventilation or the associated treatment with sedatives and muscle relaxants. Similar findings were reported in a COVID-19 study where HRQoL, assessed by EQ-5D-3L, was higher at follow-up in patients not receiving oxygen vs. invasive ventilation^20^. Unfortunately, our data cannot distinguish between the late effects of severe disease and intensive care treatment as such.

The finding that patients with previous pulmonary diagnoses regained health slower for physical functioning and physical role functioning aligns with current knowledge and the risk of impaired lung function after a severe COVID-19 infection^35^. Chronic respiratory illness is associated with an elevated risk of ICU admission in COVID-19 patients^31^. Data from a study on chronic obstructive pulmonary disease (COPD) patients treated in intensive care for exacerbation showed a decreased HRQoL compared to a reference population but not to a matched COPD cohort^36^ indicating that the lower HRQoL we see may be partly a preexisting problem caused by the underlying disease.

A similar argument can be made for the observation that higher BMI correlated with slower recovery in physical functioning, most likely because obesity is a risk factor for severe disease with ICU admission in COVID-19^31^. However, obesity has been associated with lower scores in all SF-36 dimensions, even in non-ICU patients, as recently reviewed^37^. The most marked effect of obesity is seen in physical functioning, which is consistent with our results.

Patients with diabetes mellitus had a slower recovery in general health, vitality and mental health, highlighting that the disease affects different parts of the body and aspects of life. A study on people with diabetes showed an association between mortality over time and lower RAND-36 scores as a physical composite score (comprising the four physical dimensions) and two individual physical dimensions^38^.

Finally, previous psychiatric diagnoses were associated with slower recovery in social functioning. Because anxiety disorders, including social phobia, specific phobias and generalized anxiety disorder, are the most common psychiatric conditions^39^, this effect could have been stronger, assuming that patients with psychiatric conditions were less likely to participate in the study. Additionally, these results may be affected by our exclusion criteria for mental illnesses.

### Strengths and limitations

To the best of our knowledge, our study is the first to report a multidimensional assessment of long-term HRQoL in a COVID-19 cohort of this size treated in the ICU. Another strength of the study is that we used individual RAND-36 dimensions, identifying the specific areas of affected HRQoL. Moreover, the cohort is large and most patients have been followed up personally. Also, most of the patients submitted the RAND-36 tool at a patient visit with the option of asking questions. Finally, the multicenter design increases the external validity of the study.

Because a number of potential participants died before the follow-up there is a risk of selection bias, where some more severely ill patients were not included. Similarly, bias in those not able to participate due to poor recovery or will of moving on in life is also a risk. However, the results identify risk factors of long-term reduction in HRQoL that are similar to those that are associated with more severe disease. Further, more severe disease in the form of mechanical ventilation is a risk factor for reduced HRQoL. Together this indicates that if anything this would tend to make the current analysis more conservative.

The rate of recovery may be affected by rehabilitative interventions after ICU treatment. However, in the present study we lack detailed data on access to rehabilitation and the type of interventions whether in-hospital or after discharge. The different standard of reporting rehabilitation between the cohorts and incomplete data makes this question intractable to formal analysis.

Another limitation is that the time-to-event analysis is based on random variation in follow-up times rather than continuous follow-up which leaves it susceptible to reverse causation due to selection bias and survivor bias. However, the main contributor to variation in time to follow-up was the availability of time slots for follow-up on the investigator side rather than determined by the patients. Thus, we do not believe the time-to-event analysis was strongly biased by symptom severity. Further, the results from the non-time dependent analysis and previous studies are consistent with our results lending them additional credibility. Unfortunately, the reference populations were not collected during the pandemic and although there is data showing decreased HRQoL in general population in Sweden^27^ from the pandemic period we lack RAND-36 data on it. Finally, although all patients in this study were treated in intensive care (i.e., high level of care), it is impossible to overlook the risk that patients during the pandemic may have received ICU care with lower standards, making a comparison with other non-COVID-19 ICU studies difficult. Ideally, the reference group would be collected simultaneously with the study in Finland and Sweden.

### Clinical implications

The finding of a decreased HRQoL strongly suggests patients treated in the ICU for COVID-19 perceive reduced HRQoL compared to the general population long after discharge. Moreover, patients with female sex and certain comorbidities are more prone to slow recovery. These data suggest that ICU patients, especially those with risk factors identified in this study, should be followed up to evaluate whether they benefit from rehabilitation interventions.

### Future studies

It would be useful to study a large cohort repeatedly over an extended period, given that the RAND-36 instrument is intended for longitudinal studies with a sequence of measurements and to study the long-term impact on HRQoL. Such a study would benefit from an ICU control cohort to explore different trajectories with different reasons for ICU admission. Future studies should also focus on the causes of diminished HRQoL and potential rehabilitation interventions in this group.

## Conclusion

In a cohort of patients treated in the ICU with COVID-19 HRQoL decreased compared to the general population during the first year after ICU discharge. Female sex and comorbidities were associated with a slower HRQoL recovery rate.

### Supplementary Information

Supplementary Information.

## Data Availability

Data are available through the SciLifeLab data repository after securing ethical permission and appropriate data access agreements (https://doi.org/10.17044/scilifelab.14229410), communications in these regards is referred to MH.

## Abbreviations

BMI: Body mass index
CI: Confidence interval
COPD: Chronic obstructive pulmonary disease
COVID-19: Coronavirus disease 2019
ECMO: Extra corporal membrane oxygenation
HRQoL: Health-related quality of life
ICU: Intensive care unit
IQR: Inter quartile range
SF-36: 36-Item short form survey
VAS: Visual analog scale
QoL: Quality of life
