# Supplementary material for: Health-related quality of life after surviving intensive care for COVID-19: a prospective multicenter cohort study
Source: Sci Rep. 2023 Oct 21;13:18035. doi: 10.1038/s41598-023-45346-2 (PMC10590404; doi:10.1038/s41598-023-45346-2)
Supplement: Supplementary file 1 — Supplementary Information. [file 41598_2023_45346_MOESM1_ESM.pdf]

Online supplement

Health-related quality of life after surviving intensive care for COVID-19: A prospective multicenter cohort study

Peter Halvorsen[1], Michael Hultström[1,2], Johanna Hästbacka[3,4], Ing-Marie Larsson[1], Rakel Eklund[5], Filip K Arnberg[5], Laura Hokkanen[6] Robert Frithiof[1], Ewa Wallin[1], Lotti Orwelius[7,8], Miklós Lipcsey[1,9]

1. Anesthesiology and Intensive Care Medicine, Department of Surgical Sciences, Uppsala University, Uppsala, Sweden.

2. Integrative Physiology, Department of Medical Cell Biology, Uppsala University, Uppsala, Sweden.

3. Department of Perioperative and Intensive Care Medicine, Helsinki University Hospital, and Helsinki University, Helsinki, Finland.

4. Department of Anesthesiology and Intensive Care, Tampere University Hospital and Tampere University, Tampere, Finland.

5. National Centre for Disaster Psychiatry, Department of Medical Sciences, Uppsala University, Uppsala, Sweden.

6. Department of Psychology and Logopedics, Faculty of Medicine, University of Helsinki, Helsinki, Finland.

7. Departments of Intensive Care Linköping University Hospital, Sweden.

8. Biomedical and Clinical Sciences, Linköping University, Linköping, Sweden.

9. Hedenstierna Laboratory, Department of Surgical Sciences, Uppsala University, Uppsala, Sweden.

Corresponding author:

Peter Halvorsen

peter.halvorsen@uu.se

Order of content

Exclusion criteria

Uppsala

Linköping

Helsinki

Number of patients included at the different sites

Sensitivity analyses

Exclusion criteria

The exclusion criteria vary somewhat among hospital sites because the material was collected at three sites with three separate ethical permissions and in conjunction with other studies.

Uppsala, Sweden

Exclusion criteria were <18 years of age and failure to consent. In the adjoining study, pregnancy, breastfeeding, chronic kidney failure or intoxication with substances harmful to the kidney were exclusion criteria's, not for this part, however there were none included.

Linköping, Sweden

Exclusion criteria were <18 years of age, not sufficient in the Swedish language to complete forms in Swedish or adequately communicate with the investigators.

Helsinki, Finland

Exclusion criteria were <18 years of age, not having Finnish or Swedish as native language, preexisting major neurological diagnosis (i.e. traumatic brain injury, dementia, stroke, Parkinson's disease), substantially impaired hearing or vision or developmental disability.

Number of patients included at the different sites

Uppsala included 172 patients.

Linköping included 18 patients.

Helsinki included 62 patients.

Sensitivity analyses

Here are the statistical calculations with the Swedish reference cohort as an endpoint. Please notice that there were no added or lost significances. Since we used non parametric testing we had the option to dichotomize our values at the reference level of the respective countries reference cohorts (25,26) are in the respective country that the observations were made. Since there were more observations in Sweden, we chose to use the Swedish reference data in our sensitivity analysis.

Supplement table. RAND-36 data at the first follow-up vs. the lower limit of the 95% CI in Swedish reference material (26). A two-sided binomial test was used. CI below 0.5 indicates decreased health relative to the reference.

Dimension	95% CI for 0.5	P-value	n below ref	N total
**Physical functioning**	0.317 - 0.442	<0.001	153	246
**Physical role functioning**	0.298 - 0.421	<0.001	158	246
**Bodily pain**	0.380 - 0.506	0.077	140	251
**General health**	0.250 - 0.368	<0.001	172	248
**Vitality**	0.418 - 0.546	0.611	128	247
**Social functioning**	0.365 - 0.492	<0.05	142	248
**Emotional role functioning**	0.407 - 0.534	0.375	132	249
**Mental health**	0.394 - 0.522	0.203	134	247

CI=confidence interval

Suplement figure showing Hazard ratios for the RAND-36 dimensions, including all observations in reference to the Swedish reference cohort's lower 95% CI limit (26) as the endpoint.

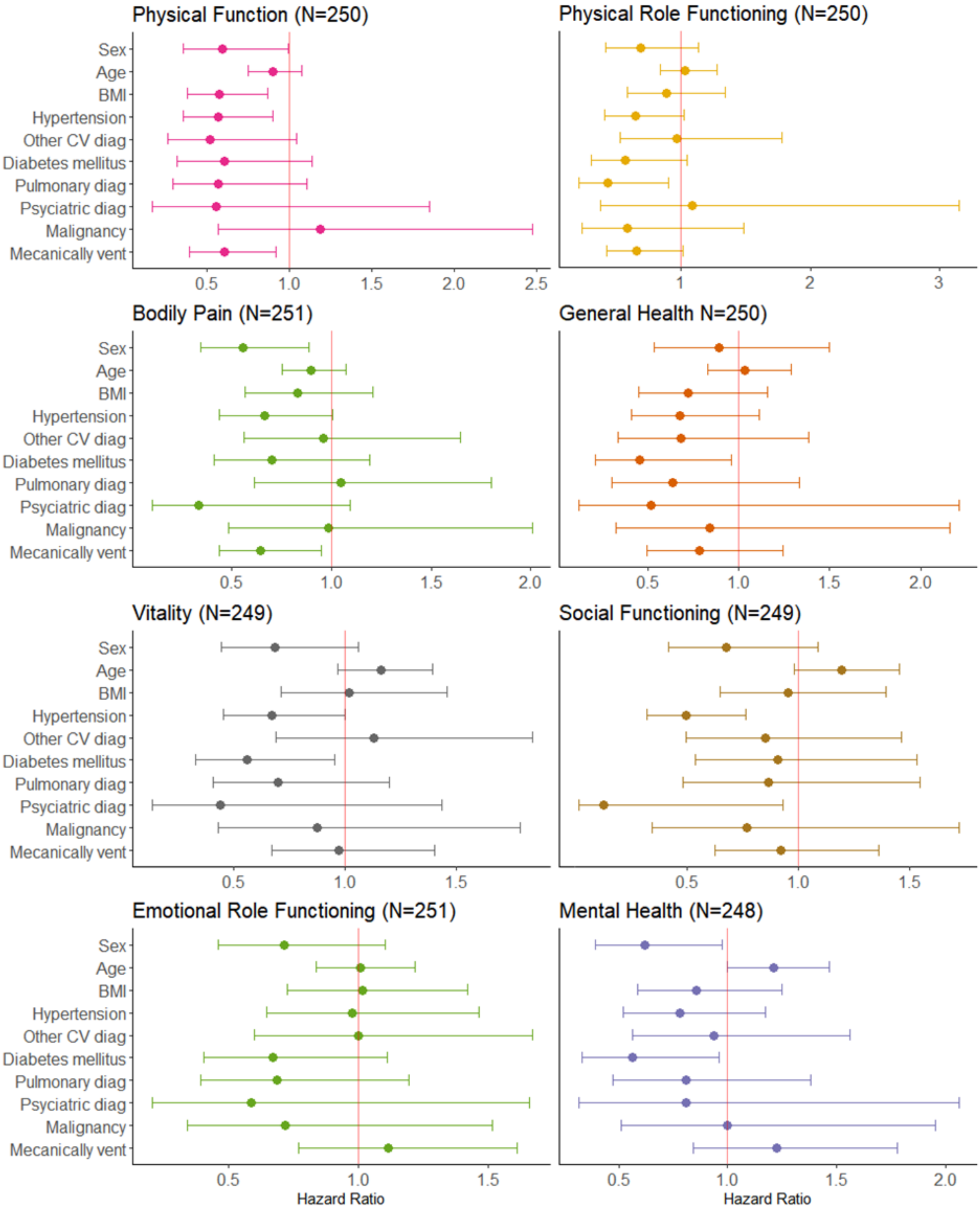

Note: Age and BMI are transformed to deca years and deca Kg/m², BMI=body mass index, CV=cardiovascular diagnoses, Sex=female.
